# Mental health and service utilization among cisgender-heterosexual, sexual minority, and gender minority autistic adults

**DOI:** 10.3389/fpsyt.2026.1766767

**Published:** 2026-02-06

**Authors:** Shalini Sivathasan, Mario J. Crown, Elizabeth Rutenberg, Sarah Brammell, Brian C. Thoma, Kelly Beck, Caitlin M. Conner, Carla A. Mazefsky, Dana Rofey

**Affiliations:** 1 Department of Counseling, Developmental, & Educational Psychology, Boston College, Boston, MA, United States; 2 Department of Occupational Therapy, Boston University, Boston, MA, United States; 3 Albert Einstein College of Medicine, New York, NY, United States; 4 Department of Psychiatry, University of Pittsburgh School of Medicine, Pittsburgh, PA, United States

**Keywords:** adulthood, autism, mental health, service utilization, sexual and gender minority

## Abstract

**Introduction:**

Autistic people are more likely than non-autistic people to identify as sexual minority (SM) and/or gender minorities (GM), yet mental health research on these intersecting identities remains underexplored.

**Methods:**

Using online self-report data from a large non-referred sample of 712 autistic adults (18–77 years), we compared GM, (cisgender) SM, and cisgender-heterosexual (CisHet) groups on sociodemographics, psychiatric diagnoses, service utilization, as well as current anxiety and depression symptoms. We also ran regression analyses to evaluate associations between sociodemographic characteristics and of lifetime psychiatric diagnosis.

**Results:**

Twenty-six percent of participants endorsed having an SM identity, and 15% reported having a GM identity. Despite high rates of psychiatric diagnoses and service use in our sample overall, SM and GM participants reported higher rates of lifetime psychiatric diagnoses, outpatient service use, psychiatric hospitalizations than CisHet participants. SM and GM groups were also more likely to report current use of psychotropic medications, with the exception of antipsychotics, which were more common in the CisHet group. Moreover, regression analyses revealed that after controlling for age, educational attainment, and sex assigned at birth, sexual and gender identity variables were most strongly associated with reported diagnostic outcomes; specifically, SM and GM participants were 2–5 times more likely to report having received depression, anxiety, PTSD, and ADHD diagnoses compared with the CisHet group.

**Conclusion:**

Our findings add to growing evidence that sexual and gender identities are critical variables to consider in autism research. We observed substantial differences in mental health outcomes across groups, underscoring the need for further investigation to better understand the diverse needs of autistic adults.

## 1 Introduction

One in 45 American adults has a documented autism diagnosis (1). Extensive research has demonstrated that autistic people experience poorer mental health outcomes (2) and face significant barriers to quality health care (3, 4), relative to non-autistic adults (3). General estimates suggest that the majority of autistic people have been diagnosed with at least one psychiatric disorder in their lifetime (70-88%) (2, 5–7), rates that are starkly higher than those of the non-autistic adults broadly (around 20%) (4, 8–10). While reasons for these elevated psychiatric challenges are multifaceted (2), emerging evidence suggests that certain subgroups within the autistic population may be at particularly heightened risk, including those who identify as LGBTQIA +.^^1^*^.

A growing literature has established that autistic adults are significantly more likely to endorse a LGBTQIA+ identity than are non-autistic individuals (11–14). Prior studies have found that 10-52% of autistic people endorse a sexual minority (SM) identity and 4-23% endorse a gender minority (GM) identity (11, 14–17), compared with 2-15% and 0.5-4.5% among non-autistic individuals, respectively (18, 19). It is also well established that LGBTQIA+ adults experience significantly higher rates of mental health challenges, including depression, anxiety, PTSD, and suicidal ideation, compared to cisgender heterosexual (CisHet) individuals (20–23).

Despite the high prevalence of LGBTQIA+ identities among autistic people, little research has explicitly evaluated psychiatric diagnosis and service use in this population. A recent methodological scoping review of studies on gender diversity and autism found that among 84 quantitative, qualitative, and mixed-methods studies, only seven studies included a mental health focus as part of their investigation (24). Results within this subset of studies found significantly higher depression and anxiety levels (25), greater lifetime self-harm and suicidal behaviors (26), and greater likelihood of PTSD (27) among individuals with LGBTQIA+ identities and autism diagnosis or traits compared to cisgender or non-autistic individuals. In the UK, another study identified significant positive associations between gender and experiences of autistic minority stress and related masking behaviors on current depression, anxiety, and posttraumatic stress symptoms (28). McQuaid and colleagues (16) examined SM identity and anxiety and depression symptoms among 651 US autistic adults recruited through the Simons Powering Autism Research (SPARK) online research registry, and found that SM-identified autistic adults reported greater anxiety and depression symptoms compared with heterosexual autistic adults. Exploratory analyses revealed that participants with a GM identity reported even higher anxiety and depression scores. While this finding did not survive correction for multiple comparisons due to the small GM sample size, the authors noted its clinical importance and emphasized the need for future research to examine these potential associations directly.

Despite growing evidence of elevated mental health challenges among LGBTQIA+ autistic adults, critical gaps remain. Most notably, none of the aforementioned studies have compared specific psychiatric diagnosis rates across SM-, GM-, and CisHet autistic adult groups. This represents an important limitation given that diagnosis count can be a key indicator of the overall mental health burden (29). Similarly, limited information exists on psychiatric service use patterns across these groups in the autistic adult population. Hall and colleagues (30) reported in a preliminary study of health disparities among people with disabilities in the US that LGBTQIA+ autistic adults were more than twice as likely to self-report a psychiatric condition, experience higher rates of unmet healthcare needs, not have their health insurance accepted, and be refused services by a medical provider, compared with non-LGBTQIA+ autistic adults. Similarly, Wallisch et al. (17) found that American LGBTQIA+ autistic adults reported significantly more days of poorer mental health and greater unmet healthcare needs than CisHet autistic adults. Though these preliminary findings provide insight into mental health service experiences at the intersection of autism and LGBTQIA+ identities broadly, more specificity in studies with larger samples is needed to understand service use trends among SM, GM, and CisHet autistic adults.

Finally, understanding how sexual and gender identities, in combination with other sociodemographic factors, are associated with psychiatric diagnoses is a critical step toward identifying those at greatest risk. Yet, this relationship remains underexplored in autistic populations. For example, people assigned female sex at birth (AFAB) are more likely to identify with LGBTQIA+ identities (31) and to receive psychiatric diagnoses, particularly anxiety, depression, and PTSD (32–34). AFAB individuals have historically been underrepresented within the autism research literature (7), often receiving autism diagnoses years later than people assigned male at birth (AMAB) (35) and frequently after other psychiatric disorders have been diagnosed (36). Given these patterns and the frequent conflation of sex and gender in autism literature (37) explicitly examining relative associations between sex and gender, among other sociodemographic variables, and mental health outcomes among CisHet and LGBTQIA+ autistic adults is essential.

Through this study we aimed to: 1) compare psychiatric diagnoses, current symptoms, and service utilization, and 2) assess the relative contribution of sociodemographic characteristics (i.e., age, educational attainment, assigned sex) on the likelihood (presence or absence) of endorsing a particular psychiatric diagnosis among three distinct groups: CisHet, SM, and GM autistic adults.

We hypothesized that SM and GM autistic individuals would report more pronounced mental health challenges (i.e., more psychiatric diagnoses, greater anxiety and depression symptoms) than CisHet autistic individuals, with GM autistic adults experiencing the greatest burden. We also hypothesized that SM and GM autistic adults would have lower psychiatric service utilization (i.e., behavioral services and medication use) compared to CisHet autistic adults. Finally, we examined whether sexual and gender identity group membership (CisHet, SM, GM), along with other sociodemographic characteristics (age, sex assigned at birth, and educational attainment), would predict lifetime psychiatric diagnoses, though we did not formulate *a priori* hypotheses about the relative strength of these associations. While we acknowledge the considerable heterogeneity within these subgroups, we focused this secondary analysis on broader SM and GM categories, to build foundational knowledge in this emerging area of autism research, and to maintain adequate statistical power, as LGBTQIA+ participants were not specifically recruited during original study recruitment.

## 2 Method

Data came from a larger online psychometric study on aspects of adult life among individuals with autism and/or intellectual developmental disabilities (38, 39). Study procedures were approved by the University of Pittsburgh Institutional Review Board. Autistic adults were recruited through the Simons Powering Autism Research (SPARK) (40) research registry (n=570) and regional organizations (n=142). For the current study, participant data were pulled from two sources: the sociodemographic survey and two measures of current anxiety and depression. See **Supplementary Materials** online and in Conner et al (38) and MacKenzie et al (39) for more information.

### 2.1 Sociodemographics

From the sociodemographic survey, we extracted data on participant-reported sociodemographic characteristics (i.e., age, sex assigned at birth, gender and sexual identity, ethnoracial identity, personal income, education, and employment), as well as psychiatric history and service utilization. See below for details on variable manipulation for the grouping variable, and refer to the **Supplementary Materials** online for other sociodemographic variables.

#### 2.1.1 Grouping variable

See **Table 1** for variables used to create the comparison groups. In alignment with LGBTQIA+ research/language conventions, participants were categorized into the CisHet group if their assigned sex and gender identity were both male or both female, and their selected sexual identity was straight/heterosexual. Participants were categorized in the SM group if their assigned sex and gender identity were both male or both female (i.e., cisgender), and their selected sexual identity was any identity other than straight/heterosexual. Finally, participants were categorized in the GM group if their assigned sex and gender identity were male and female (i.e., transfeminine), or female and male (i.e., transmasculine), or if they selected another gender identity (e.g., non-binary/gender fluid; see **Table 1** for full list of options). Participants who selected intersex were also included in the GM group.

**Table 1:** Variables used in creating the comparison groups.

Variable	Response options
Sex assigned at birth	Measured using a “select one” multiple-choice question with the following options: Female; Male; Intersex; Unsure; Prefer not to answer.
Gender identity	Measured using a “select one” multiple-choice question with the following options: Female; Male; Nonbinary/Gender Fluid; Agender/No Gender; Unsure/Questioning; Other; Prefer not to answer.
Sexual identity/orientation	Measured using a “select one” multiple-choice question with the following options: Lesbian; Gay; Bisexual; Pansexual; Queer; Straight/Heterosexual; Asexual/Aromantic; Unsure/Questioning; Other; Prefer not to answer; I don’t know.

Of note, participants did not have the option of specifying “cisgender” or “transgender” woman or man in this study, and therefore these identities were deduced by analyzing alignment or discrepancy in responses to sex and gender survey questions. Additionally, three participants who identified as GM and heterosexual were excluded to maintain focus on the intersectional experiences of gender minorities who are also sexual minorities, ensuring clearer interpretability when comparing with (cisgender) SM and CisHet groups.

#### 2.1.2 Psychiatric history

Respondents were asked, “Have you ever been diagnosed with [psychiatric diagnosis]”? Data were extracted for the following diagnoses of interest for the current study: depression, anxiety disorders, PTSD, ADHD, bipolar disorder, and obsessive-compulsive disorder (OCD). For each diagnosis, participants indicated whether they had received the diagnosis currently or in the past (collapsed into “lifetime history”).

#### 2.1.3 Service utilization

We analyzed three aspects of available data on service utilization: lifetime type of outpatient service(s) (e.g., individual, behavioral, group), lifetime frequency of psychiatric hospitalizations, and current types of psychotropic medication(s) used.

### 2.2 Current anxiety and depression symptoms

Available data were analyzed from the Patient-Reported Outcomes Measurement Information System-Emotional Distress, Short Form (PROMIS-SF) Anxiety and Depression Scales (41, 42). Respondents rated four self-report items over the past seven days (e.g., “I felt worthless”) using a 5-point Likert scale (1=“Never” to 5=“Always”). Total T-Scores ≤55 are “within normal limits,” T-Scores 55–60 indicated “mild impairment,” and >60 indicated “moderate impairment” (i.e., clinically significant symptoms). Both PROMIS-SF measures are validated for use with autistic adults and demonstrate excellent internal consistency compared with population norms (PROMIS-SF Anxiety: α=0.94, PROMIS-SF Depression: α=0.95) (43). Cronbach alphas for the current study sample were similarly strong (Anxiety: α=0.90; Depression: α=0.91).

### 2.3 Data analytic plan

Using SPSS version 29, basic descriptive analyses (chi square, ANOVA, and *post hoc* tests) were first used to assess sociodemographic characteristics among the full sample and to compare across the three groups of interest (GM, SM, and CisHet autistic participants), as well as to analyze psychiatric history and service use patterns (Aim 1). Next, binary logistic regressions were used to assess the potential influence of sociodemographic characteristics on participant-reported psychiatric history (presence versus absence of individual diagnoses) (Aim 2). Predictor variables were entered in a stepwise fashion: age and educational attainment (step 1), assigned sex (step 2), and comparison group (step 3). Bonferroni corrections were employed resulting in statistical significance achieved at *p* <.0125.

## 3 Results

### 3.1 Sociodemographic characteristics

#### 3.1.1 Sex, gender, and sexual identity among autistic participants

Data on assigned sex, gender, and sexual identity (used to create the three comparison groups) were available for *N* = 712 participants (see **Table 2**). Fifty-nine percent of participants comprised the CisHet group (*n* = 422) and 41% endorsed an LGBTQIA+ identity: 26% of the study sample comprised the (cisgender) SM group (*n* = 183), and 15% comprised the GM group (*n* = 107).

**Table 2:** Participant characteristics (total sample and by group).

Sociodemographic characteristics	Total study sample	Sexual and gender identity groups			χ^2^ or *F*	*p*	$\eta_{p}^{2}$ *or V*
	(*N* = 712)	GM (*n* = 107)	SM (*n* = 183)	CisHet (*n* = 422)	*(df)*		
Age, years (mean, SD)	35.23 (12.59)	31.8 (11.4)	34.5 (10.7)	36.4 (13.5)	6.26 (2)	.002*	.017
Assigned sex, n (%)							
Male	297 (41.7%)	24 (22.4%)	51 (27.9%)	222 (52.6%)	49.81 (2)	<.0001*	.27
Female	412 (57.9%)	80 (74.8%)	132 (72.1%)	200 (47.4%)			
Intersex	3 (0.4%)	3 (2.8%)	–	–	–	–	–
Gender identity, n (%)							
^a^Male (Man)	289 (40.6%)	16 (15.0%)	51 (27.9%)	222 (52.6%)	35.77 (2)	<.0001*	.24
^a^Female (Woman)	340 (47.8%)	8 (7.5%)	132 (72.1%)	200 (47.4%)			
Nonbinary/Gender Fluid	57 (8.0%)	57 (53.3%)	–	–	–	–	–
Agender/No Gender	5 (0.7%)	5 (4.7%)	–	–	–	–	–
Unsure/Questioning	18 (2.5%)	18 (16.8%)	–	–	–	–	–
Another gender identity	3 (0.4%)	3 (2.8%)	–	–	–	–	–
Sexual identity, n (%)							
Heterosexual/Straight	422 (59.3%)	–	–	422 (100%)	–	–	–
Lesbian	25 (3.5%)	7 (6.5%)	18 (9.8%)	–	.93 (1)	.34	.057
Gay	17 (4.2%)	6 (5.6%)	11 (6.0%)	–	.20 (1)	.89	.89
Bisexual	78 (11%)	23 (21.5%)	55 (30.1%)	–	2.52 (1)	.11	.093
Pansexual	55 (7.7%)	27 (25.2%)	28 (13.3%)	–	4.34 (1)	.04*	.12
Queer	28 (3.9%)	18 (16.8%)	10 (5.5%)	–	9.99 (1)	.002*	.19
Asexual/Aromantic	62 (8.7%)	17 (15.9%)	45 (24.6%)	–	3.04 (1)	.08	.081
Unsure/Questioning	19 (2.7%)	7 (6.6%)	12 (6.6%)	–	.00 (1)	1.00	.000
Another sexual identity	6 (0.8%)	2 (1.9%)	4 (2.2%)	–	.03 (1)	.86	.011
^b^Ethnoracial identity, n (%)							
White Non-Hispanic/Latine	568 (79.8%)	85 (79.4%)	145 (79.2%)	338 (80.1%)	5.77 (12)	.93	.065
Black/African American	39 (5.5%)	6 (5.6%)	9 (4.9%)	24 (5.7%)			
American Indian/Alaska Native (AIAN)	36 (5.1%)	6 (5.6%)	10 (5.5%)	20 (4.7%)			
Asian American/Pacific Islander (AAPI)	18 (2.5%)	4 (3.7%)	4 (2.2%)	10 (2.4%)			
White Hispanic/Latine	33 (4.6%)	4 (3.7%)	10 (5.5%)	19 (4.5%)			
Hispanic/Latine only (no race)	8 (1.1%)	0 (0.0%)	1 (0.5%)	7 (1.7%)			
Something else	10 (1.4%)	2 (1.9%)	4 (2.2%)	4 (0.9%)			
^c^Educational attainment, n (%)							
Up to and including high school graduate	143 (20.1%)	16 (15.0%)	28 (15.3%)	99 (23.5%)	7.44 (2)	.02*	.10
Some college/technical school or Associate’s degree	274 (38.5%)	53 (49.5%)	67 (36.6%)	154 (36.6%)	6.43 (2)	.04*	.095
Bachelor’s degree	152 (21.4%)	23 (21.5%)	47 (25.7%)	82 (19.5%)	2.92 (2)	.23	.064
Some post-graduate education/degree	142 (20.0%)	15 (14.0%)	41 (22.4%)	86 (20.4%)	3.11 (2)	.21	.066
Income, n (%)							
Less than $20,999	280 (42.5%)	53 (51.5%)	67 (40.1%)	160 (41.1%)	15.25 (12)	.23	.108
$21,000 to $35,999	153 (23.2%)	28 (27.2%)	35 (21.0%)	90 (23.1%)			
$36,000 to $50,999	76 (11.5%)	9 (8.7%)	21 (12.6%)	46 (11.8%)			
$51,000 to $65,999	53 (8.0%)	8 (7.8%)	18 (10.8%)	27 (6.9%)			
$66,000 to $80,999	30 (4.6%)	2 (1.9%)	7 (4.2%)	21 (5.4%)			
$81,000 to $100,999	25 (3.8%)	1 (1.0%)	8 (4.8%)	16 (4.1%)			
$101,000+	42 (6.4%)	2 (1.9%)	11 (6.6%)	29 (7.5%)			
^c^Employed-Yes, n (%)	406 (57.2%)	57 (53.3%)	103 (56.6%)	246 (58.4%)	0.96 (2)	.62	.037

^a^Participants selected “male” or “female” on the sociodemographic survey question associated with gender, however, we include “man” and “woman” in parentheses to distinguish from responses to the question on assigned sex. In the GM group, selecting “male” is indicative of transmasculine identity and selecting “female,” is indicative of transfeminine identity. ^b^Ethnoracial identity was determined by creating mutually exclusive racial/ethnic categories for group comparison purposes. ^c^Educational attainment *n* = 711; Employment (part or full time), *n* = 710, see **Supplementary Information** for more detail on variable manipulation; *p <.05.

#### 3.1.2 Sex

Approximately 58% of the current study sample identified their assigned sex as female (AFAB), 42% as male (AMAB), and <1% as intersex. Approximately half of the CisHet participants identified as AFAB and half as AMAB. Conversely, both SM (*X*^2^(1)=31.55, *p* <.0001) and GM (*X*^2^(1)=29.23, *p* <.0001) participants were three times more likely to identify their sex as AFAB than AMAB, with the remaining 2.8% of the GM group identifying as intersex. The SM and GM groups did not differ in ratio of AFAB to AMAB.

#### 3.1.3 Gender

Approximately half of the study sample identified their gender as female (woman), 41% as male (man), and 11% as nonbinary/gender fluid, agender/no gender, unsure/questioning, or another gender. Whereas man to woman gender ratios were approximately 1:1 and 1:3 for the CisHet and SM groups respectively, the gender identity most selected in the GM group was nonbinary/gender fluid, followed by participants who selected unsure/questioning, men, women, agender/no gender, or another gender (see **Table 2**).

#### 3.1.4 Sexual identity

Fifty-nine percent of participants identified their sexual identity as heterosexual (i.e., the CisHet group). The SM and GM groups did not statistically differ in their rates of the following sexual identities: bisexual, asexual or aromantic, lesbian, unsure/questioning, gay, or other. A greater proportion of the GM group identified as pansexual or queer relative to the SM group (see **Table 2**).

#### 3.1.5 Additional sociodemographic characteristics

Overall, participants’ ages ranged from 18–77 years (*M* = 35.23 years). The GM group was five years younger on average than the CisHet group (p<.001); there were no significant age differences between the SM group and CisHet or GM groups. The GM group also reported higher rates of attaining some college/technical school training than either the SM (p=.031) or CisHet groups (p=.014). There were no significant group differences in terms of ethnoracial identity (80% White and Non-Hispanic/Latine), income distribution (approximately half of the sample made less than $35,999 annually), employment status (57% were employed part or full time), or recruitment source (80% of participants from each group came from SPARK, and 20% from regional recruitment sources).

### 3.2 Aim 1: self-reported psychiatric history, symptoms, and service utilization

#### 3.2.1 Lifetime psychiatric diagnostic history

Data for each of the following psychiatric diagnoses were available for 93-97% of the participants across the three groups (cases were excluded if participants selected “not sure”). The number of reported diagnoses was also summed to generate a total number of psychiatric diagnoses per participant. Overall, participants endorsed an average of 2.84 lifetime psychiatric diagnoses each, from most to least common being anxiety, depression, ADHD, PTSD, OCD, and finally bipolar disorder. See **Table 3** for a summary of findings.

**Table 3:** Psychiatric diagnoses, service use, and symptom severity (total sample and by group).

Psychiatric diagnoses and service use	Total study sample	Sexual and gender identity groups			χ^2^ or *F*	*p*	$\eta_{p}^{2}$ *or V*
	(*N* = 712)	GM (*n* = 107)	SM (*n* = 183)	CisHet (*n* = 422)	*(df)*		
# of Lifetime Psychiatric Dx, mean (SD) n=710	2.84 (1.73)	3.48 (1.56)	3.32 (1.55)	2.47 (1.75)	25.65 (2)	<.0001*	.068
Diagnostic History-Yes, n (%)							
Anxiety Disorder, n=684	533 (77.9%)	96 (92.3%)	159 (88.8%)	278 (69.3%)	42.11 (2)	<.0001*	.248
Depression, n=687	483 (70.3%)	93 (88.6%)	147 (82.1%)	243 (60.3%)	48.09 (2)	<.0001*	.265
ADHD, n=688	341 (49.6%)	60 (57.7%)	98 (54.7%)	183 (45.2%)	7.78 (2)	.020*	.106
PTSD, n=691	255 (36.9%)	53 (51.5%)	86 (48.0%)	116 (28.4%)	31.73 (2)	<.0001*	.214
OCD, n=679	186 (27.4%)	31 (29.8%)	53 (30.1%)	102 (25.6%)	1.63 (2)	.44	.049
Bipolar Disorder, n=692	143 (20.7%)	26 (24.5%)	45 (25.4%)	72 (17.6%)	5.75 (2)	.06	.091
Service History, n (%), n=712							
Individual services	531 (74.6%)	90 (84.1%)	142 (77.6%)	299 (70.9%)	9.10 (2)	.011*	.113
Behavioral services	224 (31.5%)	39 (36.4%)	59 (32.2%)	126 (29.9%)	1.79 (2)	.41	.050
Group services	188 (26.4%)	40 (37.4%)	52 (28.4%)	96 (22.7%)	9.92 (2)	.007*	.118
Social services	182 (25.6%)	29 (27.1%)	43 (23.5%)	110 (26.1%)	0.6 (2)	.74	.029
Applied behavior analysis	54 (7.6%)	6 (5.6%)	12 (6.6%)	36 (8.5%)	1.41 (2)	.49	.045
In home services	67 (9.4%)	14 (13.1%)	17 (9.3%)	36 (8.5%)	2.08 (2)	.35	.054
Psychotropic Medication Use Current-Yes, n (%)							
Antipsychotics, n=440	90 (20.5%)	14 (17.7%)	15 (12.6%)	61 (25.2%)	8.23 (2)	.016*	.137
Antidepressants, n=442	308 (69.7%)	59 (76.6%)	89 (73.0%)	160 (65.8%)	4.07 (2)	.13	.096
Mood Stabilizers, n=437	143 (32.7%)	26 (32.5%)	37 (32.2%)	80 (33.1%)	.03 (2)	.99	.008
Psychostimulants, n=441	126 (28.6%)	26 (32.9%)	38 (28.9%)	65 (27.0%)	1.04 (2)	.60	.049
Psychiatric Hospitalization History-Yes, n (%) n=712	292 (41.3%)	57 (53.3%)	83 (45.4%)	154 (36.5%)	11.59 (2)	.003*	.128
PROMIS Measures-Current (T-score mean, SD)							
Anxiety, n=712	59.92 (9.56)	62.86 (8.30)	60.54 (8.87)	58.90 (9.99)	7.98 (2, 709)	.0004*	.022
Depression, n=711	57.44 (9.64)	61.40 (8.38)	57.57 (9.38)	56.39 (9.81)	11.78 (2, 708)	<.0001*	.032

*p <.05.

Despite the high prevalence of reported psychiatric diagnoses overall, there was variability at the group level. Participants in both GM and SM groups, which did not differ from one another, reported more psychiatric diagnoses than the CisHet group (both *ps* <.0001). Specifically, CisHet participants reported significantly lower rates of anxiety, depression, ADHD, and PTSD diagnoses compared to the SM and GM groups, which had consistently comparable rates across diagnoses (i.e., anxiety: 88-82%; depression: 82-89%; ADHD: 54-58%; PTSD: 48-52%; all *ps*>.05). There were no differences in rates of OCD or bipolar disorder across the three groups, which were the least commonly reported diagnoses.

#### 3.2.2 Current anxiety and depression symptoms

PROMIS-SF Anxiety and Depression scores approached clinical cutoffs overall (T-Scores>60). GM participants scored significantly higher than SM and CisHet participants (anxiety: ps<.05, depression: ps<.01), who did not differ. Of note, the GM group’s mean scores exceeded clinical cutoffs on both scales (moderate impairment), whereas SM and CisHet means fell at or below cutoffs (mild impairment range).

#### 3.2.3 Psychiatric service utilization

##### 3.2.3.1 Lifetime outpatient psychiatric service utilization

History of outpatient psychiatric service use data was available for 97-98% of participants across the three groups. Nearly all participants (93%) had endorsed accessing outpatient service in their lifetimes. Interestingly, the GM group was significantly *more* likely to utilize individual (*X*^2^(1)=1.79, *p* = .005) services than the CisHet group (GM: 84%, SM: 78%, CisHet: 71%; see **Table 3**). The same was true for endorsement of group-based services (*X*^2^(1)=9.57, *p* = .003) (GM: 37%, SM: 28%, CisHet: 23%). There were no significant differences between either group and the SM group. Finally, there were no group differences in the use of outpatient behavioral, social, ABA or in-home services.

##### 3.2.3.2 Lifetime psychiatric hospitalizations

Forty-one percent of the overall sample endorsed at least one psychiatric hospitalization in their lifetimes. However, rates of hospitalization were significantly higher for the GM and SM groups (not significantly different from one another) than they were for the CisHet group (*ps* <.05).

##### 3.2.3.3 Current psychotropic medication use

Forty-eight percent of participants reported currently using at least one type of psychotropic medication. There were no group differences in antidepressant, mood stabilizer, or psychostimulant use between the groups. However, the CisHet group was more likely to report antipsychotic medication use than the SM group, *X*^2^(1)=7.62, *p* = .006, while the GM group usage did not differ from either of the two groups.

### 3.3 Aim 2: variables associated with lifetime psychiatric diagnosis

#### 3.3.1 Lifetime psychiatric diagnostic history

Final binary logistic regression models predicting psychiatric diagnostic history (presence vs. absence) are displayed in **Table 4**.

**Table 4:** Final binary logistic regression models predicting presence of lifetime diagnoses.

Final Regression Model Per Diagnosis	B	SE	Wald	df	p	Odds ratio (OR)	OR 95% CI
Depression Diagnosis, n=683							
Age	.024	.008	8.53	1	.003*	1.02	[1.01, 1.04]
Education-Some College (Ref: High School)	.22	.25	.81	1	.37	1.25	[.77, 2.02]
Education-Bachelor’s degree (Ref: High School)	-.24	.27	.78	1	.38	.78	[.46, 1.34]
Education-Post-Grad (Ref: High School)	.014	.31	.002	1	.97	1.01	[.55, 1.86]
Assigned Sex (Ref: Female)	-.56	.18	9.41	1	.002*	.57	[.40,.82]
SM Group (Ref: CisHet)	1.06	.23	21.65	1	<.0001*	2.90	[1.85, 4.54]
GM Group (Ref: CisHet)	1.56	.33	22.10	1	<.0001*	4.78	[2.49, 9.18]
Anxiety Diagnosis, n=680							
Age	.012	.009	1.80	1	.18	1.01	[1.00, 1.03]
Education-Some College (Ref: High School)	.018	.26	.005	1	.95	1.02	[.61, 1.69]
Education-Bachelor’s degree (Ref: High School)	.19	.31	.36	1	.55	1.20	[.66, 2.21]
Education-Post-Grad (Ref: High School)	.071	.33	.046	1	.83	1.07	[.56, 2.06]
Assigned Sex (Ref: Female)	-.80	.20	15.75	1	<.001*	.45	[.30,.67]
SM Group (Ref: CisHet)	1.09	.27	16.62	1	<.001*	2.98	[1.76, 5.05]
GM Group (Ref: CisHet)	1.65	.42	15.81	1	<.001*	5.22	[2.31, 11.80]
PTSD Diagnosis, n=687							
Age	.031	.007	17.89	1	<.001*	1.03	[1.02. 1.05]
Education-Some College (Ref: High School)	.049	.24	.042	1	.84	1.05	[.66, 1.68]
Education-Bachelor’s degree (Ref: High School)	-.50	.28	3.29	1	.070	.61	[.35, 1.04]
Education-Post-Grad (Ref: High School)	-.64	.30	4.54	1	.033	.53	[.30,.95]
Assigned Sex (Ref: Female)	-.95	.18	26.75	1	<.001*	.39	[.27,.55]
SM Group (Ref: CisHet)	.79	.20	15.74	1	<.001*	2.19	[1.49, 3.23]
GM Group (Ref: CisHet)	.85	.24	12.25	1	<.001*	2.35	[1.46, 3.78]
ADHD Diagnosis, n=684							
Age	-.001	.007	.032	1	.86	1.00	[.99, 1.01]
Education-Some College (Ref: High School)	-.25	.22	1.30	1	.25	.78	[.51, 1.20]
Education-Bachelor’s degree (Ref: High School)	-.27	.25	1.19	1	.28	.76	[.47, 1.24]
Education-Post-Grad (Ref: High School)	-.66	.28	5.66	1	.017	.52	[.30,.89]
Assigned Sex (Ref: Female)	.36	.17	4.61	1	.032	1.43	[1.03, 1.98]
SM Group (Ref: CisHet)	.49	.19	6.70	1	.010*	1.63	[1.13, 2.35]
GM Group (Ref: CisHet)	.59	.24	6.20	1	.013	1.80	[1.13, 2.85]

*p< .05

##### 3.3.1.1 Depression diagnosis

The full regression model was statistically significant, *X*^2^(7)=69.41, *p* <.0001, and explained 15% (Nagelkerke *R*^2^) of variance in depression diagnosis (see **Table 4**). When all variables were considered in the full model, age, sex, and group remained significant predictors. Increasing age was associated with greater likelihood of a depression diagnosis, and AFAB identity was 0.57 times more likely than AMAB to be associated with depression diagnosis. However, the greatest predictor was group membership. Specifically, after accounting for age, sex, and educational attainment (not a significant predictor), participants in the SM group and GM groups were 2.9 and 4.8 times more likely to report a lifetime depression diagnosis than CisHet participants, respectively.

##### 3.3.1.2 Anxiety diagnosis

Comparable findings were observed with lifetime anxiety diagnosis. The full regression model was statistically significant, *X*^2^(7)=76.01, *p* <.0001, explaining 15% of variance in anxiety diagnoses. While age and educational attainment were not significant predictors in this model, AFAB identity was 0.45 times more likely than AMAB identity to predict anxiety diagnosis. However, SM and GM participants were 2.9 and 5.2 times more likely to be diagnosed with anxiety than CisHet participants, respectively.

##### 3.3.1.3 PTSD diagnosis

The full regression model was statistically significant, *X*^2^(7)=79.87, *p* <.0001, once again explaining 15% of variance in PTSD diagnosis. Age was positively associated with likelihood of PTSD diagnosis, while educational attainment was not a significant predictor following Bonferroni corrections. SM and GM groups were 2.2 and 2.4 times more likely to be diagnosed with PTSD than CisHet participants, respectively.

##### 3.3.1.4 ADHD diagnosis

The full regression model was statistically significant, *X*^2^(7)=21.76, *p* = .003, explaining 4.2% of variance in ADHD diagnosis. However, following Bonferroni correction, the only variable associated with ADHD diagnosis was group. Specifically, the SM group was 1.6 times more likely to be diagnosed with ADHD than CisHet participants.

##### 3.3.1.5 Bipolar and OCD diagnoses

The full regression models for bipolar disorder and OCD diagnoses were not statistically significant following Bonferroni corrections, *X*^2^(7)=16.57, *p* = .02 and *X*^2^(7)=14.96, *p* = .037, respectively.

## 4 Discussion

The present study assessed psychiatric history, current symptoms, and service use patterns among SM-, GM-, and CisHet-identifying autistic adults in a secondary analysis of a large non-referred cohort (Aim 1). In our sample, 41% reported an SM (26%) or GM (15%) identity, aligning with recent findings that two in five of autistic adults endorse an LGBTQIA+ identity (14, 16, 17). Furthermore, 75% of SM and GM participants identified as AFAB, compared to just under half of participants in the CisHet group, highlighting the substantial overlap between AFAB status and LGBTQIA+ identities *within* the autistic population, and the necessity in capturing, rather than assuming, participants’ assigned sex, gender, and sexual identity in autism research and clinical practice.

Findings from Aim 1 revealed that while autistic adults are already more likely than non-autistic adults to receive psychiatric diagnoses, this is further heightened among LGBTQIA+ autistic people. Specifically, for lifetime diagnoses of depression, anxiety, PTSD, and ADHD, both SM and GM groups reported significantly higher rates than the CisHet group. Interestingly, for current symptom severity, the GM group reported higher levels of anxiety and depression than both SM and CisHet groups, which did not differ. These patterns reveal an important distinction: that SM and GM autistic adults report comparably elevated rates of lifetime psychiatric diagnoses, extending prior work that has primarily focused on current symptom severity often among SM or GM autistic adults separately (16, 25–28). However, when examining current symptoms, GM individuals report greater symptom burden on anxiety and depression measures than SM individuals, consistent with McQuaid et al. (16) The variability of SM outcomes (sometimes aligning with CisHet and sometimes with GM groups) may reflect differences in identity visibility, as sexual orientation can often be concealed while gender identity may be more apparent, though more research is needed to clarify the mechanisms underlying these differential patterns.

Similar patterns emerged for lifetime mental health service use. GM and SM groups were more likely than the CisHet group to report using some types of outpatient care, psychiatric hospitalization, and current psychotropic medication use (except for antipsychotics which CisHet participants used more). These findings extend prior research focusing on unmet healthcare needs more broadly by increasing specificity on the types of service and psychotropic medications used by each of the three groups (17, 30). It is important to note, however, that participants recruited through research registries may be more connected to services than the general autistic population. It remains unclear whether our findings of elevated service use overall stem from greater mental health needs, appropriate care, treatment barriers, or some combination (9, 44). Prescribing patterns may also vary; for example, antipsychotics are more commonly prescribed to autistic men while antidepressants are more common for autistic women (45), which could partially explain higher antipsychotic use in our CisHet group (50% AMAB-AFAB) versus the predominantly AFAB SM and GM groups.

This study also addressed a significant gap in the literature by examining and quantifying, at a large scale, the specific extent to which SM and GM identities, sex assigned at birth, and other relevant sociodemographic characteristics were associated with lifetime psychiatric diagnoses in autistic adults (Aim 2). Regression analyses revealed that group membership (CisHet, SM, GM) accounted for the greatest variance across diagnoses, surpassing all other variables in the models. SM and GM autistic adults were 3 and 5 times more likely, respectively, to have a lifetime anxiety or depression diagnosis, and 2 times more likely to have a PTSD diagnosis compared to CisHet autistic adults. While age and educational attainment showed significant but modest associations with certain diagnoses, and sex assigned at birth was significantly associated with depression, anxiety, and PTSD, sexual and gender identity emerged as the strongest characteristics associated with psychiatric diagnoses in our sample.

Overall, our findings underscore that sexual and gender identity are not merely demographic variables to control for, but central factors shaping mental health outcomes in autistic adults, that should be intentionally measured and incorporated in clinical assessment and in sample characterization. Our findings also demonstrate the need to disaggregate and compare subgroup data among autistic samples, which are often treated as homogenous, to better understand and address the mental health needs of LGBTQIA+ (and AFAB) autistic populations, both in research and in clinical practice.

### 4.1 Limitations and future directions

This study had several limitations that are critical to address in future research for CisHet and LGBTQIA+ autistic people. Logistically, our relatively small GM group prevented comprehensive within-group (e.g., cishet males and females) and between-group (e.g., cishet males and GM males) analyses, which could generate more insight into the nuanced mental health needs along the intersecting autism and LGBTQIA+ spectrums. We are also cognizant that in making our grouping determinations (CisHet, SM, GM), we are limiting our understanding of within-group heterogeneity (e.g., among individuals who endorse a gender minority identity and as heterosexual). We also lacked detailed information about service use (e.g., quality and quantity), limiting our ability to determine context for elevated service use. Future research examining the nature of service use within and between autistic participant groups would provide rich and nuanced information on mental healthcare experiences among service users. Additionally, corroborating self-reported mental health and service use rates with clinician ratings or data from electronic medical records could be helpful when considering and comparing our total sample’s rates to those of other studies.

In terms of sample characteristics, our predominantly White, highly educated sample limits generalizability, as autistic LGBTQIA+ individuals from ethnoracially and socioeconomically marginalized communities, as potential disparities might differ or be more pronounced among these populations. Moreover, our findings may not be generalizable to individuals who have less access to educational and healthcare resources. Future autism research must intentionally seek ways to racially and socioeconomically diversify recruitment so as to not overstate findings that may not be as applicable in these communities, who often face compounded barriers and/or have distinct needs.

Finally, our data were cross-sectional and self-reported, precluding causal inferences about increased psychiatric symptom risk among LGBTQIA+ autistic adults. Indeed, minority stress frameworks emphasize the multiple and compounding pathways through which minoritized groups may experience greater mental health burden. Future research should explore the mechanisms that may underlie elevated psychiatric symptoms in LGBTQIA+ autistic adults, including potential roles of stigma, discrimination, and healthcare access barriers. Additionally, future research should consider the potential contributions of late autism diagnosis (as well as misdiagnosis) to mental health experiences, particularly among AFAB and LGBTQIA+ populations (35, 36, 46). Listening to and deepening collaborations with autistic communities, who have extensively shared such first-hand experiences (47, 48), is necessary to future research in this area.

## Data Availability

Publicly available datasets were analyzed in this study. This data can be found here: National Institutes of Mental Health Data Archive (NDA) https://nda.nih.gov/.

## Footnotes

1
*Although often used interchangeably, we use the terms LGBTQIA+ when referring to the collective identity, but sexual minority (SM) and gender minority (GM) to increase specificity about the specific sub-populations, when applicable. SM refers specifically to a person whose sexual identity, orientation, or behaviors differ from the heterosexual majority. GM refers specifically to a person whose gender identity or expression differs from the gender typically associated with their sex assigned at birth.*
