# Supplementary material for: Mental health and service utilization among cisgender-heterosexual, sexual minority, and gender minority autistic adults
Source: Front Psychiatry. 2026 Feb 6;17:1766767. doi: 10.3389/fpsyt.2026.1766767 (PMC12920194; doi:10.3389/fpsyt.2026.1766767)
Supplement: Supplementary file 1 [file Table1.docx]

Supplementary Material

Initial participant recruitment: Participants in this study were obtained from non-probabilistic sampling of data collection efforts for a larger psychometric study of the Relationships, Employment, Autonomy, and Life Satisfaction (REALS) Scale – Self and Proxy Reports, which recruited individuals with autism and/or intellectual developmental disability (IDD), and caregivers (described in detail in Conner et al., 2025 and MacKenzie et al., 2024). As part of that larger study, our current study sample included *N*=712 autistic adult participants (who provided self-report responses independently) who were recruited via two methods: The majority (*n*=570) of autistic adults were recruited through the Simons Powering Autism Research (SPARK) Research Match. SPARK represents the largest online research registry of individuals (and their families) living in the United States who report having a formal autism diagnosis made by a healthcare professional that matches these individuals to various research studies they can participate in (https://sparkforautism.org/discover/tags/spark-research-match/). The remaining (*n*=142) autistic adult participants in our study came from over 160 local and national autism and IDD organizations that were contacted during recruitment. A full list of measures included in the larger Conner et al. (2025) psychometric validation study can be found in the National Institutes of Mental Health Data Archive (NDA) under R01 HD 100392.

Variable manipulation: *Race & Ethnicity:* Participants were provided two separate questions to report information on their race and their ethnicity. Participants identified their race using a “select all that apply” multiple-choice question with the following options: American Indian or Alaskan Native (AIAN); Asian; Native Hawaiian or other Pacific Islander; White; Black or African American; Other. Overall, 7.9% of the sample (*n*=56) selected more than one racial identity. Participants also identified their ethnicity using a “select one” multiple-choice question with the following options: Hispanic/Latino; Not Hispanic/Latino.

For the purpose of having a single ethnoracial variable for descriptive analysis, without excluding participants who selected multiple races, we used a deterministic bridging method to assign participants reporting more than one race to the single race they identified with that had the largest count other than White (thus prioritizing their minoritized racial identities). Thus, participants who selected Black/African American in addition to any other racial identity (*n*=39) were added to the Black racial group. From the remaining pool of multi-racial participants, any participant who identified as AIAN in addition to any other racial identity (*n*=36) was added to the AIAN group. In alignment with unified Asian American and Pacific Islander (AAPI) groups, remaining Asian or Native Hawaiian or other Pacific Islander with any other racial identification were included in an AAPI group (*n*=18). Remaining individuals (*n*=10) were classified under an Other category (renamed “Something else”). Lastly, to be inclusive of multiple racial/ethnic intersectional identities, participants were subsequently categorized into one of the following additional groups: White Non-Hispanic/Latine (*n*=568); White Hispanic/Latine (*n*=33) and Hispanic/Latine (no race selected; *n*=8). Following this categorization method, we identified no statistically significant differences in ethnoracial identity across the three comparison groups (*p*=.92).

*Educational Attainment:* Participants were asked to identify their highest educational attainment using a “select one” multiple-choice question with the following options: 0=Less than 8^th^ grade; 1=Some high school; 2=Finished high school (or equivalent); 3=Some college or technical school but did not finish; 4=AA degree or technical school; 5=Bachelor’s degree (e.g., BA, BS); 6=Some post-graduate degree work (e.g., Master’s) but did not finish; 7=Post-graduate degree; 8=Do not wish to provide).

As per conventional reporting of educational attainment categories, 0-2 were collapsed into “Up to and including high school graduate”; 3-4=Some college/technical school or AA degree; 5=Bachelor’s degree”; 6-7=“Some post graduate education/degree.”

*Income:* A similar data reduction strategy was used for the personal income variable. Participants responded to a “select 1” multiple-choice question with the following options: 0=Less than $20,999; 1=$21,000-$35,999; 2=$36,000-$50,999; 3=$51,000-$65,999; 4=$66,000-$80,999; 5=$81,000-$100,999; 6=$101,000-$130,999; 7=$131,000-$160,999; 8=Over $161,000; 9=Prefer not to answer; 10=I don’t know. Categories 6-8 were collapsed into an “Over 101,000+” category to capture the n=53 (5.9%) of participants in our sample within the top personal income categories.

*Employment:* Participants were asked to identify whether they were currently employed, using a “select one” multiple-choice question with the following options: Working-Full time paid employee or self-employed; Working-Part time paid employee or self-employed; Unpaid student intern; Not working at this time-Laid off and/or looking for work; Unable to work; Retired. Participants were categorized into two groups: Options were collapsed to capture the n=406 (57.2%) of participants “Employed for Pay,” versus “Not Employed for Pay.”

As applicable, the responses “Prefer not to answer” or “I don’t know” were excluded during data reduction.

Data excluded in the current study: From the initial group of 788 independently self-reporting autistic adult participants in the larger study, complete data for sex, gender, and sexual identity were available for *N*=712 participants. That is, participant data was excluded if they selected “Prefer not to answer” or “I don’t know,” or skipped response to any of the aforementioned group categorization variables. Three (n=3) of these 76 participants who were identified as being part of the GM group (i.e., sex and gender variables were not both ‘male’ or both ‘female’) had also selected ‘straight/heterosexual’ as their sexual identity. In this case, these participants were excluded from the GM group, which we defined as participants who endorse both a sexual and gender minority identity. While we do not have a way to verify any accidental selections across the larger data collection, we are also cognizant that we unfortunately may be contributing to the erasure of individuals who endorse a gender minority identity *and* as heterosexual.
